# Personalized Medicine Transformed: ChatGPT’s Contribution to Continuous Renal Replacement Therapy Alarm Management in Intensive Care Units

**DOI:** 10.3390/jpm14030233

**Published:** 2024-02-22

**Authors:** Mohammad S. Sheikh, Charat Thongprayoon, Fawad Qureshi, Supawadee Suppadungsuk, Kianoush B. Kashani, Jing Miao, Iasmina M. Craici, Wisit Cheungpasitporn

**Affiliations:** 1 Division of Nephrology and Hypertension, Department of Medicine, Mayo Clinic, Rochester, MN 55905, USA; sheikh.mohammad@mayo.edu (M.S.S.); thongprayoon.charat@mayo.edu (C.T.); qureshi.fawad@mayo.edu (F.Q.); supwadee.sup@mahidol.ac.th (S.S.); kashani.kianoush@mayo.edu (K.B.K.); miao.jing@mayo.edu (J.M.); craici.iasmina@mayo.edu (I.M.C.); 2 Chakri Naruebodindra Medical Institute, Faculty of Medicine Ramathibodi Hospital, Mahidol University, Samut Prakan 10540, Thailand; 3 Division of Pulmonary and Critical Care Medicine, Department of Medicine, Mayo Clinic, Rochester, MN 55902, USA

**Keywords:** artificial intelligence, continuous renal replacement therapy, CRRT, CRRT alarm, continuous kidney replacement therapy, CKRT, intensive care technology, clinical decision support systems, natural language processing, NLP, nephrology, critical care, chatbot, ChatGPT

## Abstract

The accurate interpretation of CRRT machine alarms is crucial in the intensive care setting. ChatGPT, with its advanced natural language processing capabilities, has emerged as a tool that is evolving and advancing in its ability to assist with healthcare information. This study is designed to evaluate the accuracy of the ChatGPT-3.5 and ChatGPT-4 models in addressing queries related to CRRT alarm troubleshooting. This study consisted of two rounds of ChatGPT-3.5 and ChatGPT-4 responses to address 50 CRRT machine alarm questions that were carefully selected by two nephrologists in intensive care. Accuracy was determined by comparing the model responses to predetermined answer keys provided by critical care nephrologists, and consistency was determined by comparing outcomes across the two rounds. The accuracy rate of ChatGPT-3.5 was 86% and 84%, while the accuracy rate of ChatGPT-4 was 90% and 94% in the first and second rounds, respectively. The agreement between the first and second rounds of ChatGPT-3.5 was 84% with a Kappa statistic of 0.78, while the agreement of ChatGPT-4 was 92% with a Kappa statistic of 0.88. Although ChatGPT-4 tended to provide more accurate and consistent responses than ChatGPT-3.5, there was no statistically significant difference between the accuracy and agreement rate between ChatGPT-3.5 and -4. ChatGPT-4 had higher accuracy and consistency but did not achieve statistical significance. While these findings are encouraging, there is still potential for further development to achieve even greater reliability. This advancement is essential for ensuring the highest-quality patient care and safety standards in managing CRRT machine-related issues.

## 1. Introduction

Acute kidney injury (AKI) has emerged as a serious complication among critically ill patients, with studies citing an incidence as high as 57% in certain intensive care unit (ICU) populations [1,2,3]. When severe or rapidly progressive, AKI often warrants the provision of continuous renal replacement therapy (CRRT) to prevent life-threatening electrolyte disturbances, severe metabolic acidosis, fluid overload, and uremia [1,4,5]. CRRT involves the slow but continuous extracorporeal removal of plasma water containing solutes and toxins, which is balanced with replacement fluid to maintain homeostasis [6]. Compared to intermittent modalities, CRRT provides superior hemodynamic stability, steady solute clearance, precise fluid balance, and real-time electrolyte regulation in hemodynamically unstable patients [6,7].

Several factors influence the utilization of CRRT in ICUs, including the severity of AKI, presence of shock, degree of fluid overload, and need for gradual solute and toxin clearance [8,9]. While CRRT serves as a pivotal therapeutic modality, its application relies on complex machinery prone to disruptions [4]. Modern CRRT devices have incorporated alarm systems to enable rapid detection of hazards and prompt intervention by healthcare staff [9,10]. Alarms alert staff to problems including blood flow irregularities, filter clotting, air entrapment, improper fluid removal rates, and vascular access malfunction, among others [4,10,11]. Considering that CRRT is an uninterrupted, around-the-clock treatment with little margin for error, alarm systems and timely responses are vital for patient safety and optimal delivery of therapy [12,13]. However, excessive alarms also contribute to alarm fatigue, especially among critical care nurses who bear the maximum brunt of such alerts. Alarm fatigue, described as desensitization from repeated exposure to nonactionable alarms, can negatively impact patient care when critical alerts are inadvertently ignored or missed [11,13]. Delayed reactions to CRRT alarms go beyond mere operational glitches, setting off critical safety concerns for patients [12,13]. Such lapses may result in cardiovascular instability, as made evident through abrupt blood pressure fluctuations that compromise patient hemodynamics, which is crucial for critically ill individuals who need stable cardiovascular management. Moreover, these delays can lead to electrolyte imbalances, with significant shifts in potassium, calcium, or magnesium levels potentially causing arrhythmias or neuromuscular dysfunction [12,13]. Additionally, the effectiveness of treatment delivery suffers, thus impacting the removal of toxins and excess fluids, and may result in uncontrolled uremia, fluid overload, or acid–base imbalances. The aggregation of these problems emphasizes the critical need for immediate and efficient action in response to CRRT alarms to prevent prolonged ICU stays or increased mortality risks [13].

In recent years, artificial intelligence (AI) models have demonstrated tremendous transformative potential across diverse spheres of clinical medicine [14]. Advanced natural language processing (NLP) tools like ChatGPT-3.5 and ChatGPT-4 can comprehend complex concepts, solve problems logically, summarize lengthy text and generate insightful output [15,16,17,18,19]. These AI assistants employ cutting-edge self-supervised deep learning to accumulate formidable stores of knowledge across myriad domains. Applications within healthcare have included automated diagnosis, optimized imaging interpretation, personalized treatment plans and improved clinical workflows. However, the integration of AI in the niche domain of CRRT management remains limited and under-investigated [20]. Currently, there lies a significant gap in the literature when it comes to leveraging NLP models for optimizing CRRT practices, alarm responses, and patient safety protocols. While few studies have commented generally on AI applications in nephrology, empirical evidence evaluating model performance on deciphering CRRT alarms is lacking [20]. Most AI research has, understandably, emphasized more prevalent specialties, whereas CRRT management represents an underexplored frontier [20].

Therefore, this study aims to bridge this gap in knowledge by comparing two cutting-edge NLP models, ChatGPT-3.5 and ChatGPT-4, on a validated benchmark of CRRT alarms. The specific objectives are to critically evaluate and compare both assistants’ accuracy, consistency, and reliability in analyzing these alarms and providing appropriate solutions to address each scenario. Their responses will be scored against gold-standard solutions crafted by domain experts to enable standardized assessments. Thereafter, their performance will be quantified across pertinent metrics, and current limitations, as well as opportunities for enhancements, will be highlighted. By undertaking this pioneering research at the intersection of artificial intelligence and nephrology, this study hopes to provide greater clarity on translating such tools to advance patient safety and therapeutic effectiveness in the administration of CRRT worldwide [21].

## 2. Materials and Methods

### 2.1. Data Collection

**Figure 1:**
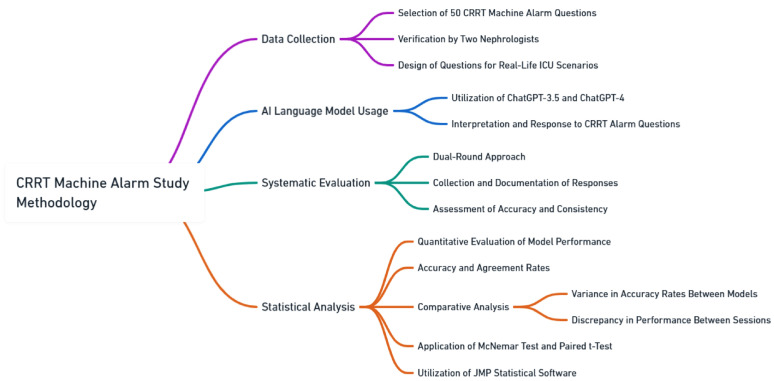
Study flow diagram.

In the context of this study, our primary data collection revolved around addressing a set of 50 questions related to CRRT machine alarms. These questions were not randomly selected; rather, they underwent a meticulous selection and verification process by two nephrologists in intensive care. This careful curation was pivotal, ensuring that the questions represented a wide spectrum of scenarios frequently encountered in ICU environments. The design of each question was intentional, aiming to reflect the nature of real-life clinical queries. This approach was adopted to ensure that the responses elicited from the AI models would have practical value and applicability in real-world ICU settings (Figure 1).

### 2.2. AI Language Model Usage

We utilized the capabilities of ChatGPT-3.5 and ChatGPT-4, models representing the cutting-edge of OpenAI’s NLP advancements [22]. These systems are underpinned by state-of-the-art NLP algorithms that build on machine learning approaches—especially deep learning and transformers—and are fine-tuned to emulate human-like language understanding and respond with impressive accuracy. In our study, we specifically focused on evaluating the accuracy of ChatGPT-3.5 and ChatGPT-4 [22] in providing responses to CRRT alarm questions (Online Supplementary Materials).

### 2.3. Systematic Evaluation

The evaluative phase of the study was executed in December 2023. Both the ChatGPT-3.5 and ChatGPT-4 models were engaged in two separate sessions to address the curated CRRT machine alarm questions. This dual-round approach was strategic, serving multiple objectives. Primarily, it allowed for the collection and documentation of the models’ responses, forming a robust dataset for subsequent analysis. A crucial part of the evaluation involved assessing the models’ performance in two key areas: accuracy and consistency. The accuracy metric was determined by juxtaposing the responses from ChatGPT-3.5 and ChatGPT-4 against a predefined answer key, which was meticulously crafted by the two nephrologists in intensive care. Consistency, on the other hand, was gauged by examining the coherence between the responses from the two separate sessions for each model. This metric provided valuable insights into the stability and reliability of the models’ performance over repetitive tasks.

### 2.4. Statistical Analysis

The analytical framework of the study was designed to present a clear, quantitative evaluation of the models’ performance. The results, particularly accuracy and agreement rates, were quantified and expressed as counts with corresponding percentages. A comparative analysis was conducted to explore two dimensions: the variance in accuracy rates between ChatGPT-3.5 and ChatGPT-4 and the discrepancy in performance between the first and second session for each model. The McNemar test was the statistical tool of choice for this comparison, providing a robust framework for analyzing paired nominal data. Further, the mean accuracy score for each ChatGPT version was computed by averaging the scores from the two sessions. These mean scores were then compared between ChatGPT-3.5 and ChatGPT-4, employing a paired *t*-test to determine the statistical significance of any observed differences. For the purpose of this study, a two-sided *p*-value of less than 0.05 was established as the threshold for statistical significance. All statistical computations and analyses were facilitated using the JMP statistical software (version 16; SAS Institute, Cary, NC, USA), a choice that was driven by the software’s comprehensive capabilities and robust analytical tools.

## 3. Results

Table 1 shows the accuracy and agreement of ChatGPT-3.5 and -4’s responses to CRRT machine alarm questions. Out of 50 questions, ChatGPT-3.5 correctly provided 43 (86%) and 42 (84%) responses in the first and second runs, respectively.

**Figure 2:**
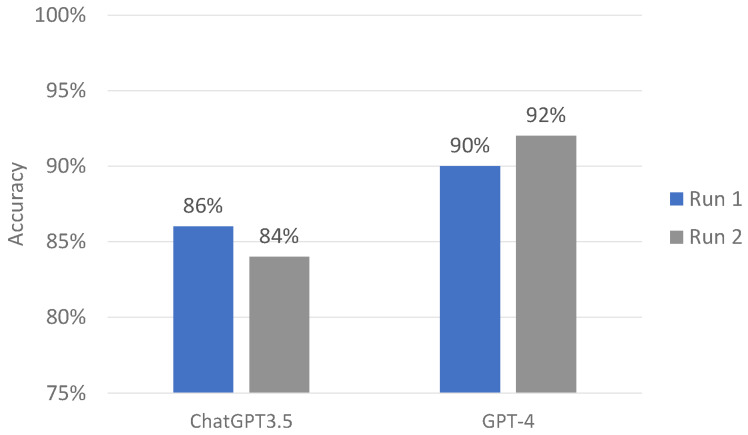
ChatGPT-3.5 and GPT-4 performance on CRRT alarm questions.

The mean accuracy score of ChatGPT-3.5 was 85%. ChatGPT-3.5 consistently provided 42 (84%) responses between the first and second runs with the Kappa statistic of 0.78. In contrast, ChatGPT-4 correctly offered 45 (90%) and 47 (94%) responses in the first and second runs, respectively. The mean accuracy score of ChatGPT-4 was 92% (Figure 2).

ChatGPT-4 consistently provided 46 (92%) responses between the first and second run with the Kappa statistic of 0.88. Although all of performance parameters in term of accuracy and consistency of ChatGPT-4 was higher than those of ChatGPT-3.5, the difference in these parameters between ChatGPT-3.5 and -4 was not statistically significant. In evaluating open-ended queries and narrative responses, both iterations of ChatGPT demonstrated consistency in their responses, aligning with the established multiple-choice answer key while avoiding the generation of recommendations that could be considered detrimental.

## 4. Discussion

The integration of AI into healthcare, particularly within the realm of critical care, heralds a transformative era that promises to enhance patient safety and clinical efficiency significantly. AI’s utility in healthcare is multifaceted, spanning predictive analytics, patient triage, and decision support systems. AI has the potential to improve several parts of CRRT care [23]. It could help recognize patients needing CRRT sooner, estimate patient survival odds and kidney recovery chances, and identify risk factors related to dying [24]. Some research has explored using machine learning to upgrade CRRT, as well. Studies look at monitoring CRRT in real time, foreseeing CRRT problems, and gathering data to review at the bedside. Other work has used algorithms to predict if blood pressure drops when starting CRRT. This highlights how AI could boost understanding complications and outcomes for CRRT patients. AI and machine learning can likely make providing CRRT much safer and more effective moving forward [24]. As these systems keep improving, they may become extremely helpful for managing this intensive ICU therapy [23,24].

The rapid data processing capabilities of AI models, such as ChatGPT, are indispensable in the high-pressure, data-intensive environments characteristic of critical care, where every decision is time-sensitive and informed by myriad complex datapoints. Clinicians in these settings are often inundated with a plethora of alarms, many of which may be false or noncritical, leading to a phenomenon known as alarm fatigue [25]. This phenomenon is a significant concern in healthcare, as it can desensitize providers to alerts, potentially slowing response times or causing critical alarms to be overlooked. Therefore, managing alarms effectively is critical, particularly in the context of CRRT, where the accurate interpretation of machine alarms is essential for patient safety and the effectiveness of treatment [26]. The complex nature of CRRT machinery and the critical importance of correctly interpreting its alarms underline the potential life-or-death implications of any misinterpretation [27,28,29,30].

This study’s comparative analysis of ChatGPT-3.5 and ChatGPT-4 in the specific context of addressing CRRT machine alarm questions sheds light on the progressive evolution of AI capabilities in healthcare. The findings indicate a marked improvement in both accuracy and consistency from ChatGPT-3.5 to ChatGPT-4, reflecting the rapid advancements in natural language processing and AI technologies. The observed increase in accuracy rates (from 86.0% to 90.0% in the first run and 84.0% to 92% in the second run) from ChatGPT-3.5 to ChatGPT-4 is significant. It highlights the models’ enhanced ability to understand and process specialized medical language, which is a crucial competency in critical care settings such as CRRT alarm management. The high accuracy rates of both models, especially ChatGPT-4, are promising indicators for their potential future applications in healthcare. However, these high levels of accuracy also emphasize the continued need for supervision and verification by medical professionals, considering the high-stakes nature of CRRT management [31].

The transformative potential of AI in revolutionizing CRRT is vast, offering advancements in quality assurance, risk prediction, and bedside decision-making [24,32]. However, the integration of AI into CRRT practices is still in its nascent stages. It is characterized by a limited evidence base and concerns about potential biases in data sources and algorithmic designs [33]. The research directed toward harnessing AI to optimize CRRT delivery is burgeoning, yet the field is in a developmental phase, calling for more comprehensive investigations and validations. This is particularly crucial given the potential for AI-induced disparities in healthcare provision. Despite AI’s recognized potential in transforming healthcare, a significant research gap exists in its application to specific tasks such as CRRT alarm troubleshooting. Our study pioneers in expanding the application domain of AI within healthcare, moving beyond conventional patient data analysis to encompass operational aspects of healthcare. It delves into the complex realm of interpreting and responding to alarms from specialized medical devices like CRRT machines, signifying a broadening of AI’s role in healthcare to include both patient-centric and operational dimensions of medical care [34]. This research is particularly relevant for ICUs around the world, in which intensivists often manage CRRT. Our findings show that AI can make CRRT management more effective, thus helping the healthcare team work better and make quicker, more informed decisions. This could greatly assist intensivists in ICUs, thus improving patient care. By improving how alarms are handled and supporting faster decision-making, AI has the potential to enhance the work of doctors and nurses significantly. Our study not only explores new technological applications in healthcare but also demonstrates how these advancements can aid medical professionals daily, ultimately benefiting patient care.

The consistency of model responses, as reflected by Cohen’s kappa statistic, is a critical aspect of this study. The higher kappa value for ChatGPT-4 compared to ChatGPT-3.5 (0.889 vs. 0.759) indicates a more reliable and steady performance across repeated trials, which is an extremely important feature in medical uses in which inconsistency could lead to varied patient outcomes [35]. Therefore, the higher kappa value for ChatGPT-4 indicates a significant step forward in making AI tools more dependable for clinical applications. Additionally, the study underscores the models’ proficiency in generating narrative responses and adeptly managing open-ended questions, which is a skill that is highly relevant in medical settings in which complex language and the need for thorough clarifications are common [36]. The ability of both ChatGPT versions to align with a multiple-choice answer key without leading to potentially dangerous recommendations showcases the models’ robust understanding and interpretation of complex medical scenarios.

Our study explored whether large language models (LLMs) like ChatGPT can assist with CRRT machine alarms [20]. We aimed to evaluate ChatGPT’s ability to generalize across various CRRT devices by testing it with a range of sample alarms and issues. However, ChatGPT does not connect to or control CRRT machines. Rather, its knowledge comes from analyzing technical manuals, troubleshooting guides, and clinical examples for these devices. By training on this diverse material, ChatGPT can provide recommendations for resolving alarms on CRRT machines that it has not directly encountered before. Our key motivation is finding innovative ways that artificial intelligence might ease alarm fatigue for healthcare workers [34]. Excessive clinical alarms lead to stress and disrupt patient care. If systems like ChatGPT can help streamline responses to minor device alerts, providers could focus more attention on patients. Although it is in an early stage, we believe exploring how AI might enhance clinical workflows is worthwhile. As AI and LLMs continue advancing, they will likely take on more responsibilities in healthcare. This trend matters for our work because it shows how AI might simplify and boost medical decision-making. We want to apply AI to reduce the flood of alarms overwhelming intensive care units [34]. Too many alarms can desensitize staff and hurt patient care. If systems like ChatGPT can competently handle basic machine alerts, caregivers could better focus on patients. Our results around ChatGPT managing continuous renal replacement therapy device alarms suggest that AI could lessen burdens that healthcare workers face from alarms. This opens the door to future tools that make alarm responses more efficient, free up clinician time, and improve care quality and safety.

However, it is crucial to acknowledge the inherent limitations of AI tools like ChatGPT in healthcare applications [37]. The potential for errors, although minimal, underscores the necessity for continuous improvement and human oversight [38,39]. The performance of these AI models is heavily contingent on the quality and specificity of the input data, highlighting the importance of well-rounded training datasets that encompass a diverse array of medical scenarios and terminologies. Furthermore, the study’s focus on a specific type of medical equipment, CRRT machines, may not fully encapsulate the models’ capabilities in other medical contexts [39,40]. The reliance on predetermined answer keys for accuracy assessment may not accurately reflect the complex decision-making required in real-world clinical settings [41]. Additionally, while the results are promising, they are derived from a controlled experimental setting and may not directly translate to clinical practice without further empirical validation [42]. Our study takes a careful approach to applying AI in critical medical areas like RRT, which is a stance that underscores our commitment to patient safety and a medical professional’s oversight [43]. This initial examination of how AI can aid in troubleshooting alarms from CRRT machines marks the start of a broader exploration. We openly acknowledge the study’s limited scope and the necessity for additional research before expanding AI implementation here. As we continue, our aims are multipronged: conducting robust studies to address current restrictions and hurdles; gathering more expansive data; refining algorithms to handle complex medical situations better; and boosting the efficiency of working alongside caregivers. This integration must center on improving care while preserving irreplaceable human guidance. More research is required, but prudently applied AI could someday amplify CRRT safety and effectiveness.

Future research should aim to apply AI models like ChatGPT in a broader spectrum of medical equipment and scenarios, including real-time clinical applications [44]. Exploring the integration of AI tools into actual patient care would offer more comprehensive insights into their practical utility and limitations. The ramifications of such advancements could be profound, potentially ushering in a new era of enhanced, precise, and efficient medical care, particularly in high-stakes environments such as critical care. Improving patient monitoring and medication safety with technology involves acknowledging the potential benefits and challenges. Continuous improvement, validation, and oversight are essential to upholding patient care standards. Effectiveness hinges on the quality of the data that these technologies are trained on. Comprehensive datasets covering various medical scenarios and focusing on patient safety and medication accuracy are crucial. This approach reduces adverse events and bolsters the reliability of healthcare technology. In addition, future research should look beyond just OpenAI’s GPT-4 to examine a wider range of LLMs. This should include open-source options such as Falcon, LLaMA, and Guanaco-65B, as well as proprietary models like Claude and PaLM 2 [20]. Each model has unique strengths, from Falcon’s language breadth and Guanaco-65B’s memory efficiency to Claude’s ethical foundations and PaLM 2’s advanced reasoning. Studying these diverse LLMs could greatly help use AI to enhance medical care precision, efficiency, and results. Potential benefits span improved patient monitoring, medication safety, and overall reliability but require continuous upgrades, extensive validation, and strict governance. Success will largely hinge on training datasets that represent an array of clinical scenarios focused on patient wellbeing and treatment accuracy. Expanding the LLMs assessed will unlock new AI potential to drive healthcare advancement and real-world applications.

Integrating AI like LLMs for CRRT alarm handling has immense potential to enhance healthcare efficiency, accuracy, and outcomes [43]. However, thoughtfully addressing critical policy and ethical considerations is vital during implementation. Regulatory frameworks must establish standards ensuring that these technologies are applied safely, effectively, and equitably. Guidelines on data quality, transparency, and accountability should protect patients and prevent bias, necessitating collaboration between healthcare leaders, ethicists, and policymakers to navigate complex environments. Ethical usage requires informed consent, upholding patient rights and trust, with clarity for clinicians and patients on AI tool use, including benefits vs. limitations. It also means ensuring equitable access so that AI does not worsen disparities. Additionally, AI should augment clinician capabilities, not override human judgment for high-stakes decisions. Policies should promote AI as an assistive rather than decisive role, keeping care teams engaged. Managing such risks needs strong encryption, access controls, monitoring, high-quality training data, and having clinicians review AI suggestions before using them.

Developing, deploying, and governing AI instruments for CRRT necessitates collaborative attempts [24,26]. These attempts need participation between technologists, nephrologists, critical care experts, health policy professionals, patients, and other pivotal voices [24]. Such cross-disciplinary cooperations can generate AI solutions that are cutting-edge yet clinically valuable, ethically grounded, and truly incorporated into real-world care, thereby driving substantive enhancements for patients. Regulatory entities play an integral role by instituting standards around safety, efficacy, privacy, and bias reduction that are paramount for preserving trust. Meanwhile, healthcare organizations’ ethical committees assess the moral implications of employing AI in patient care, emphasizing consent and prudent use given the intricate ethical issues in critical care settings. This multidimensional approach seeks to ensure AI innovations meet benchmarks. The highest benchmarks are for care quality and ethical integrity, ultimately advancing outcomes in CRRT and other vital care areas.

## 5. Conclusions

The study’s findings are a step forward in integrating AI into healthcare, particularly in critical care settings. The improved performance of ChatGPT-4 over ChatGPT-3.5 in CRRT alarm troubleshooting indicates a forward trajectory in AI development. While these advancements are promising, continuous development and rigorous validation are required to ensure reliability and safety in clinical applications. Incorporating AI into healthcare is filled with potential, but it must be undertaken with a careful understanding of its limitations and the utmost emphasis on patient safety.

## Figures and Tables

**Table 1:** The accuracy and agreement of ChatGPT-3.5 and -4 in answering CRRT machine alarm questions.

	ChatGPT-3.5	ChatGPT-4	*p*-Value
Accuracy—1st run	43/50 (86)	45/50 (90%)	0.63
Accuarcy—2nd run	42/50 (84)	47/50 (94%)	0.18
Average accuracy	85%	92%	0.09
Agreement	42/50 (84%)	46/50 (92%)	0.34
Kappa statistics	0.78	0.88	-

Accuracy—1st run and 2nd run: represents the proportion of questions correctly answered by ChatGPT versions 3.5 and 4 during the first and second rounds of testing, respectively. Average accuracy: calculated as the mean accuracy across both runs for each ChatGPT version.

## Data Availability

The article’s data will be shared with the corresponding author at a reasonable request.

## Supplementary Materials

The following supporting information can be downloaded at: https://www.mdpi.com/article/10.3390/jpm14030233/s1, CRRT Questions: Alarms and Troubleshooting.
