# Supplementary material for: Personalized Medicine Transformed: ChatGPT’s Contribution to Continuous Renal Replacement Therapy Alarm Management in Intensive Care Units
Source: J Pers Med. 2024 Feb 22;14(3):233. doi: 10.3390/jpm14030233 (PMC10971480; doi:10.3390/jpm14030233)
Supplement: Supplementary file 1 [file jpm-14-00233-s001.zip › jpm-2859624-supplementary.pdf]

**Online Supplementary**

CRRT Questions: Alarms and Troubleshooting

1. What is the recommended action to address a "low arterial pressure alarm" during continuous renal replacement therapy (CRRT)?
   a. Increase blood flow rate
   b. Administer antibiotics
   c. Remove kinks or clamps in the access line
   d. Switch to a different access device
2. What action should be taken when the CRRT machine alarms " LOW ARTERIAL PRESSURE ALARM" and found to have access device against vessel wall?
   a. Increase the set blood flow rate
   b. Declamp the kinked line
   c. Switch to a different access device
   d. Administer anticoagulants
3. How should you respond to a "Return Pressure Positive" alarm in CRRT Circuit?
   a. Increase the blood pump rate
   b. Decrease the blood pump rate
   c. Check for obstruction in the return line
   d. Increase the replacement pump rate
4. What action is advised when the CRRT effluent pressure reads +200 mmHg?
   a. Increase the blood pump rate
   b. Check for kinks in the effluent line
   c. Increase the replacement pump rate
   d. Decrease the blood pump rate
5. What should you do if the CRRT machine alarms "Filter Clotting"?
   a. Increase the anticoagulation rate
   b. Notify the physician
   c. Replace the circuit
   d. Continue therapy as is
6. The CRRT machine alarms "Blood Leak" during CRRT therapy. You should:
   a. Increase the anticoagulation
   b. Notify the physician immediately

    c. Ignore the alarm
    d. Replace the effluent bag
7. The CRRT machine alarms "Air in Blood" after changing the CRRT circuit. You should:
    a. Increase the anticoagulation rate
    b. Locate and purge the air
    c. Notify biomed of a machine failure
    d. Continue therapy
8. The replacement fluid pump stops running during CRRT. You should:
    a. Replace the hemofilter
    b. Obtain a new replacement bag and spike it
    c. Decrease the blood pump rate
    d. Notify the physician
9. The CRRT machine alarms "Scales Open". You should:
    a. Make sure all scale levers are fully closed
    b. Notify biomed of a malfunction
    c. Check the return line for obstruction
    d. Increase the replacement pump rate
10. What action should be taken if the effluent bag fills rapidly during CRRT?
    a. Notify the physician
    b. Check for obstruction in the effluent line
    c. Increase the dialysate rate
    d. Draw a laboratory ammonia level
11. The replacement fluid bag runs dry during CRRT. You should:
    a. Obtain new replacement fluid and spike it
    b. Increase the dialysate rate
    c. Decrease the blood pump rate
    d. Notify biomed of a pump failure
12. The dialysate solution bag runs dry during CRRT. You should:
    a. Obtain new dialysate and spike it
    b. Turn off the machine and change circuits
    c. Increase the replacement pump rate
    d. Notify the physician of a shortage

13. The CRRT machine alarms "Return Pressure Negative". You should:
    a. Decrease the blood pump rate
    b. Check for leaks in the return line
    c. Increase the replacement pump rate
    d. Change the hemofilter
14. The replacement fluid contains air bubbles during CRRT. You should:
    a. Increase the anticoagulation rate
    b. Decrease the replacement pump rate
    c. Stop therapy and purge the air
    d. Notify the physician
15. The CRRT machine alarms "Filter Clogging." You should:
    a. Increase the anticoagulation
    b. Continue therapy
    c. Notify the physician
    d. Replace the patient's catheter
16. What action should be taken if the effluent bag overflows during CRRT. You should:
    a. Press the mute button and continue therapy
    b. Empty the effluent bag
    c. Decrease all pump rates
    d. Notify biomed of a machine failure
17. The CRRT machine alarms "Access Disconnected". You should:
    a. Reconnect the access line
    b. Increase the replacement pump rate
    c. Change the circuit
    d. Draw a complete blood count
18. The dialysate solution pump rate increases spontaneously during CRRT. You should:
    a. Reset the dialysate pump rate
    b. Notify biomed of a malfunction
    c. Increase the replacement pump rate
    d. Continue therapy
19. The CRRT machine alarms "Return Disconnected". You should:
    a. Reconnect the return line

b. Decrease the blood pump rate
c. Check the access line
d. Flush the return catheter

20. The replacement fluid pump is alarming during CRRT. You should:
    a. Reset the replacement pump rate
    b. Change the circuit
    c. Decrease the dialysate pump rate
    d. Notify the physician
21. How should you address a "blood pump speed too slow" alarm in continuous renal replacement therapy?
    a. Increase the set blood flow rate
    b. Decrease the blood flow rate
    c. Stop the session
    d. Replace the entire circuit
22. What action should be taken if HIGH VENOUS PRESSURE ALARM and found to have "clotted line" occurs during continuous renal replacement therapy?
    a. Increase the blood pump speed
    b. Replace the entire circuit
    c. Declot the access
    d. Wait for the machine to recalibrate
23. The CRRT machine alarms "Effluent Volume Full" during continuous renal replacement therapy. You should:
    a. Empty the effluent bag
    b. Increase the replacement rate
    c. Notify biomed of a malfunction
    d. Clamp the effluent line
24. The CRRT machine alarms "Replacement Volume Empty" during continuous renal replacement therapy. You should:
    a. Hang a new replacement fluid bag
    b. Notify the pharmacy
    c. Keep running until dayshift
    d. Decrease the blood pump rate

25. The CRRT machine alarms "Access Disconnected" when moving the patient. You should:
    a. Reconnect the access line
    b. Check the patient's central line
    c. Increase the replacement rate
    d. Decrease the blood pump rate
26. The effluent pressure on the CRRT machine reads -150 mmHg. You should:
    a. Notify biomed of malfunction
    b. Check for kinks in the effluent line
    c. Increase the replacement rate
    d. Change the circuit
27. The CRRT machine alarms "Access Pressure Negative" *after filter change*. You should:
    a. Increase the blood pump rate
    b. Check for access line kinks
    c. Decrease the replacement rate
    d. Notify biomed of malfunction
28. The CRRT machine alarms "Unmeasurable Access Pressure". You should:
    a. Change the vascular access
    b. Check line connections and restart
    c. Decrease the blood pump rate
    d. Notify biomed of malfunction
29. The CRRT machine alarms "High Venous Pressure". You should:
    a. Increase the dose of diuretics
    b. Decrease the blood pump rate
    c. Change the circuit
    d. Check for central venous stenosis
30. The CRRT machine alarms "Unmeasurable Return Pressure". You should:
    a. Check line connections and restart
    b. Notify biomed of malfunction
    c. Place patient in Trendelenburg
    d. Increase the replacement rate
31. What should you do if the CRRT machine continues to alarm with the message “access extremely negative:

a. Check the patient’s position and machine’s access line
b. Increase the machine's blood pump speed
c. Administer additional anticoagulants
d. Discontinue the CRRT session immediately

32. What steps should be taken if the CRRT machine would not run despite changing bags?
    a. Verify the new bags are correctly hung and check for any machine errors
    b. Disconnect the patient from the machine and wait for spontaneous recovery
    c. Clean the machine's exterior surfaces
    d. Speak to the machine in a soothing voice
33. What is the normal pressure range for the effluent line of CRRT circuit?
    a. 0 to 1000
    b. -200 to -300
    c. +50 to +150
    d. 42 to 97
34. What is the normal pressure range for the red-coded access line of CRRT circuit?
    a. +200 to +300
    b. 50 to -150
    c. 0 to 100
    d. d. 42 to 97
35. What is the normal pressure range for the blue-coded return line of CRRT circuit?
    a. +50 to +150
    b. b. -200 to -300
    c. c. 0 to 1000
    d. d. 42 to 97

36. What Should you do if the CRRT machine alarms after changing the effluent bag?
    a. Wait for the machine to self-correct
    b. Check the effluent line for any obstructions or kinks
    c. Consult the machine's user manual for troubleshooting
    d. Calculate the machine's expected lifespan in hours
37. What should you do if the CRRT machine alarms during a procedure or a trip?
    a. Examine the machine's exterior for hidden buttons

b. Check if the machine needs a software update
c. Pause the procedure and investigate the cause of the alarm
d. Evaluate the machine's screen for hidden settings

38. What should you do if the CRRT machine alarms and the patient is agitated?
    a. Perform a system reboot on the machine
    b. Examine the machine's tubing for kinks or blockages
    c. Offer the patient a calming conversation
    d. First stabilize the patient’s condition, then investigate the cause of the alarm

39. What should you do if the CRRT machine alarms and you are in the middle of drawing labs?
    a. Run a self-diagnostic test on the machine
    b. Pause the lab draw and resolve the alarm before continuing
    c. Check the machine's screen for error codes
    d. Ask the patient to remain still during the lab draw

40. What should you do if the CRRT machine alarms while changing the patient’s position?
    a. Check the access and return lines for any kinks or obstructions
    b. Adjust the machine's alarm sensitivity
    c. Calibrate the machine's pressure sensors
    d. Briefly pause the procedure to ensure the machine's stability

41. What should be done if the CRRT machine alarms during a bath procedure for a patient?
    a. Verify the machine's water temperature settings
    b. Pause the bath and investigate the cause of the alarm
    c. Perform a machine software update
    d. Engage the patient in a conversation about their comfort

42. What should be the first step if the CRRT machine alarm reads “Scale Open”?
    a. Clear the alarm and attempt to restart the machine
    b. Inspect the machine's tubing for blockages
    c. Check if the machine's filters need replacement
    d. Consult the machine's maintenance schedule

43. What should you do if the CRRT machine alarms while you are hanging a new dialysate bag?
    a. Review the machine's fluid flow settings
    b. Verify that the new bag is correctly hung and connected

c. Run a diagnostic check on the machine's fluid management system

d. Discuss the machine's operation with a colleague

44. How should you respond to a "disconnection alarm" during continuous renal replacement therapy?
    a. Check the circuit and patient for disconnection
    b. Declamp the line if it's kinked or clamped
    c. Increase the set blood flow rate
    d. Evaluate for a circuit change

45. When experiencing a "fluid balance error" alarm during continuous renal replacement therapy, what should you do?
    a. Wait for bags to stabilize
    b. Increase the machine's blood pump speed
    c. Remove kinks in effluent bags
    d. Change the machine immediately

46. What is the correct response to a "turbulence close to sensor" alarm during continuous renal replacement therapy?
    a. Increase the machine's blood pump speed
    b. Override the alarm
    c. Check the circuit for kinks
    d. Follow instructions for degassing

47. How should you address a "machine systemic error" alarm during continuous renal replacement therapy?
    a. Increase the machine's speed
    b. Change the entire circuit
    c. Replace the machine with a new one
    d. Call technical assistance and do not reuse the machine before their response

48. What is the primary cause of a "disconnection alarm" during continuous renal replacement therapy?
    a. Presence of air bubbles in the circuit
    b. A kink in the return line
    c. Line separation or disconnection from the patient (rare)
    d. Machine malfunction

49. How should you respond to a "Positional Vascular Access Obstruction" causing a "High Venous Pressure Alarm" during CRRT?
    a. Change the machine's blood flow settings
    b. Switch to a different access device
    c. Increase the anticoagulation rate
    d. Declot the access device

50. What action should be taken if the CRRT machine alarms with a "Clot Excluding Pressure Sensor" message?
    a. Increase the blood flow rate
    b. Evaluate for a circuit change
    c. Change the machine's filter
    d. Ignore the alarm and continue therapy
